# Randomized controlled trial on promoting influenza vaccination in general practice waiting rooms

**DOI:** 10.1371/journal.pone.0192155

**Published:** 2018-02-09

**Authors:** Christophe Berkhout, Amy Willefert-Bouche, Emmanuel Chazard, Suzanna Zgorska-Maynard-Moussa, Jonathan Favre, Lieve Peremans, Grégoire Ficheur, Paul Van Royen

**Affiliations:** 1 Department of General Practice/ Family Medicine, School of Medicine, Lille University, Lille, France; 2 Department of Public Health, University Hospital Lille, EA, Lille University, Lille, France; 3 Department of Primary and Interdisciplinary Care, University Antwerp, Antwerpen, Belgium; 4 Department of Nursing and Midwifery, University Antwerp, Antwerpen, Belgium; 5 Mental health and wellbeing Research Group (MENT), Vrije Universiteit Brussel, Brussels, Belgium; University of Washington, UNITED STATES

## Abstract

**Background:**

Most of general practitioners (GPs) use advertising in their waiting rooms for patient’s education purposes. Patients vaccinated against seasonal influenza have been gradually lessening. The objective of this trial was to assess the effect of an advertising campaign for influenza vaccination using posters and pamphlets in GPs’ waiting rooms.

**Methods and findings:**

Registry based 2/1 cluster randomized controlled trial, a cluster gathering the enlisted patients of 75 GPs aged over 16 years. The trial, run during the 2014–2015 influenza vaccination campaign, compared patient’s awareness from being in 50 GPs’ standard waiting rooms (control group) versus that of waiting in 25 rooms from GPs who had received and exposed pamphlets and one poster on influenza vaccine (intervention group), in addition to standard mandatory information. The main outcome was the number of vaccination units delivered in pharmacies. Data were extracted from the SIAM-ERASME claim database of the Health Insurance Fund of Lille-Douai (France). The association between the intervention (yes/no) and the main outcome was assessed through a generalized estimating equation. Seventy-five GPs enrolled 10,597 patients over 65 years or suffering from long lasting diseases (intervention/control as of 3781/6816 patients) from October 15, 2014 to February 28, 2015. No difference was found regarding the number of influenza vaccination units delivered (Relative Risk (RR) = 1.01; 95% Confidence interval: 0.97 to 1.05; *p = 0*.*561*).

**Conclusion:**

Effects of the monothematic campaign promoting vaccination against influenza using a poster and pamphlets exposed in GPs’ waiting rooms could not be demonstrated.

## Introduction

The French National Health Insurance conducts a seasonal influenza immunization campaign every year but the influenza vaccination rate has been decreasing in people over 65 years of age (from 63.3% in 2009 to 54.0% in 2011), in people under 65 suffering from diverse target diseases (from 40.3% to 33.1%) and in people over 65 with target disease (from 72.3% to 60.7%) in France as well as in many other countries, since 2009 [1]. It has never reached the national and European objective of 75% [2,3]. For this reason, public advertising has been intensified (TV, newspapers and magazines) and the involvement of health professionals was stimulated, particularly by means of encounters with Health Insurance delegates, and posters and pamphlets to be exposed in GPs’ waiting rooms.

GPs’ waiting rooms are reconsidered as educational sites in the field of prevention and health education, notably through posters and pamphlets [4–7]. These media are emphasized by their eased use and their evoking of patients’ interest. The increasing number of educational material is correlated to a larger patients’ satisfaction [8–10]. Linking a poster with pamphlets seems to improve patients’ knowledge scores and likely influence their health behaviour. The literature indicates no clear strategy in health promotion using audio-visual material in general practitioners’ waiting rooms and the effect on patient health behaviour appears to be small [6] or controversial [11,12]. If any, more than 10,000 subjects are necessary to demonstrate any effect.

This randomized clinical trial was performed to evaluate the effect of an advertising campaign using posters and pamphlets in GPs’ waiting rooms on the number of influenza vaccination units delivered in community pharmacies.

## Materials and methods

### Study design

The trial was conducted in the area of the Lille-Douai Health Insurance district (Northern France) during the institutional seasonal influenza vaccination campaign of 2014–2015.

It was a single blinded 2/1 registry-based cluster randomized controlled trial design. A computerized random draw was used to allocate GPs in each group. In the intervention group, 25 GPs received and were supposed to expose in their waiting rooms 135 pamphlets and one poster (added to the usual mandatory information) withdrawing all the other posters. In the control group, waiting rooms were kept in their usual state. Fifty GPs in the control group were not aware of the intervention but knew that the seasonal influenza vaccination uptake of their patients would be measured. The outcome was the number of seasonal influenza vaccination units released in community pharmacies. Baseline was defined as the 2013–2014 seasonal influenza vaccination campaign. Data were extracted between October 15, 2014 and February 28, 2015 from the SIAM-ERASME claim database of the Lille-Douai district Health Insurance Fund on patient level. The study was lengthened by one month (February 2015) following the extension of the vaccination campaign duration by health authorities. The protocol had therefore been amended.

The prescription of the seasonal influenza vaccine units was delivered by an individual GP depending on the skills, training and motivation of the latter. This may lead to a potential clustering of the outcome for patients treated by the same GP. To provide valid results, considering the cluster allocation by GP of patients, a correction has to be introduced in computing the number of GPs needed for the trial, called intra-cluster correlation coefficient (ICC). According to Austin PC, the largest mean difference in power for the analysis of a cluster randomized trial with binary outcomes was used, with an ICC of 0.02, for α = 5% and β = 20%.[13] With a predicted rate of influenza vaccination delivery of 0.65 in the intervention group and 0.60 in the control group, and a target size by GP of 400 patients, 75 GPs had to be enrolled (50 in the control group and 25 in the intervention group).[1]

### Target population

The GPs participating in the study had previously been contacted by telephone and had given their written consent. GPs without waiting room, or sharing one single waiting room with several other GPs, or participating in another ongoing study, were not eligible to participate in this study. Language barriers in patients were not considered.

The study population existed of patients over the age of 16, who were registered by the Health Insurance on the participating GPs’ patients lists. The target population were patients over the age of 65 or having a chronic disease requiring seasonal influenza immunization (like COPD or diabetes). Patients were informed about the anonymous use of their data and could refuse to participate at any time.

### Statistical analysis

A univariate analysis was performed first, to present the baseline characteristics of patients after randomization, respectively in the intervention and control groups. Quantitative variables were expressed as mean with a 95% Confidence Interval (95% CI) of the mean. Categorical variables were expressed as percentages and 95% CI of the percentage. The clustering variable GP was taken into account through the *svydesign* function of the package *survey* [14].

To assess the association between the vaccination status (dependent variable) and the group intervention/control, because of the hierarchical structure of the data and the high incidence rate of the main outcome, we used a generalized estimating equation (GEE) Poisson regression clustering by GP [15]; an exchangeable working correlation matrix was used. Although the trial is randomized and therefore the confounding factors are already balanced between the yes/no intervention groups, in order to ensure that the "previous influenza vaccination" factor was fully controlled, the variable “Previous influenza vaccination” was used as an adjustment variable in the model.

The analysis was carried out using R software (version 3.3.1) and the package Geepack [16].

### Ethics, regulation and redaction

The study protocol passed the Ethics Committee of the Lille University Hospital (CPP Nord Ouest IV, advice #: HP 14/51) and the National Electronic Data and Liberty Commission (CNIL, advice 1783641 v 0). It is registered on ClinicalTrials.gov (registration #: NCT03239795).

The CONSORT statement has been used to complete this article.

## Results

Seventy-five GPs were enrolled between July 2014 and September 2015. Twenty-five were allocated to the intervention group and 50 to the control group (S1 Fig). Data were collected from 10,597 patients, 7,952 over the age of 65 and 5,308 with a chronic disease (2,632 belonging to both categories). All patients were included for analysis, 3,781 in the intervention group and 6,816 in the control group.

Patients were comparable in both groups at baseline, thus during the 2013–2014 vaccination campaign (Table 1). Most patients were female (57.8), 89.1% of the patients had seen their GP and 46.2% had been vaccinated against influenza.

**Table 1:** Baseline characteristics (October 2013-February 2014) by study condition.

Characteristics	Category	Control group	Intervention group
		*(n = 6*,*816)*	*(n = 3*,*781)*
		*% [95% CI]*	*% [95% CI]*
Gender	Male	41.8 [40.2–43.5]	42.5 [39.9–45.1]
Age		69.0 [68.0–70.0]	69.4 [67.8–81.2]
Age≥ 65 years		74.8 [71.8–77.5]	74.9 [70.2–79.1]
Chronic disease		50.0 [47.2–52.8]	49.6 [45.3–54.0]
Influenza vaccination	2013–2014	46.4 [44.0–48.9]	45.6 [41.1–50.2]

**Legend:** n = numbers, 95% IC = 95% confidence interval, y = years

During the study period, groups remained comparable (Table 2), the median consultation rate per patient with his GP was 3.1. At least one medical consultation was performed for 84.6% of the patients (exposed to the intervention).

**Table 2:** Study characteristics (October 2014-February 2015) by study condition.

Characteristics	Category	Control group	Intervention group
		*(n = 6*,*816)*	*(n = 3*,*781)*
		*% [95% CI]*	*% [95% CI]*
Influenza vaccine delivery	2014–2015	49.0 [46.6–51.4]	48.9 [44.3–53.5]
	October 2014	34.1 [32.2–35.9]	33.6 [30.3–37.1]
	November 2014	12.1 [11.0–13.3]	12.6 [10.8–14.8]
	December 2014	2.2 [1.9–2.5]	2.0 [1.5–2.7]
	January 2015	0.4 [0.3–0.7]	0.3 [0.2–0.6]
	February 2015	0.0 [0.0–0.1]	0.1 [0.0–0.2]
Consultation rate (n)	During study	3.05 [2.78–3.33]	3.37 [2.97–3.77]
At least one consultation	During study	83.8 [76.6–89.0]	86.0 [75.1–92.6]
	All 2014	88.9 [83.8–92.6]	89.4 [76.8–95.5]

**Legend:** n = numbers, 95% IC = 95% confidence interval, y = years

No difference was found regarding the number of influenza vaccination units delivered (Relative Risk = 1.01; 95%CI = [0.97 to 1.05]; p = 0.561). A vaccination performed on the previous year increased revaccination probability (RR = 5.63; 95%CI: [5.21 to 6.10] p<0.001) (Table 3).

**Table 3:** Factors associated with increased vaccinations. October 2014—February 2015.

Characteristic	Relative Risk	95% CI	p
Randomization	1.01	0.97–1.05	0.561
Previous flu vaccination	5.63	5.21–6.10	< 0,001

**Legend:** 95% IC = 95% confidence interval

## Discussion

An effect of the monothematic campaign promoting vaccination against influenza using a poster and pamphlets exposed in GPs’ waiting rooms could not be demonstrated basing on the number of influenza vaccination units delivered in pharmacies.

Other studies evaluating audio-visual material in waiting rooms could not demonstrate any effect of posters and pamphlets [17–20]. A study conducted in Quebec did not demonstrate the effect of posters in waiting rooms on the number of vaccinated patients [19]. Lagorce found no effect of a poster and a pamphlet on the incitement to consult a doctor for women with bladder disorder [18]. A study performed in 1998 showed an increase of the knowledge score regarding vaccination against poliomyelitis in patients who had been exposed to a video. Reading a pamphlet did not increase the knowledge of those who had been exposed to the video [20]. No effect of posters in waiting rooms was proven on the decrease of antibiotic use in general practice [17].

Most of the previous studies that demonstrated an effect of audio-visual material (all type) in waiting rooms of GPs were of limited quality with a major risk of bias.[21] Only one Grade B randomized clinical trial published by Warner demonstrated a significant effect of video on incident sexually transmitted infection (STI) among patients visiting a STI-screening private hospital [9].

All themes used in audio-visual material do not have the same efficiency. It seems that patients remember the messages they feel concerned with.[22] The theme of vaccination is predominant in GPs’ waiting rooms.[23] Studies assessing this theme are controversial.[19,20,24]

Literature confirms that the intervention of the GP remains the main factor to improve immunization rates.[25] Patients are more receptive to messages submitted in waiting rooms if their doctor is motivated and proactive during the encounter.[11,26] His action increases the message of institutional public health organizations.[27] He can influence beliefs about vaccines, increase patient self-efficacy and facilitate the transition from intention to behaviour. The effect of posters and pamphlets on the vaccination intention, as written institutional media, is more normative.[28]

### Limits

The perspective of the influenza vaccination campaign was considered from a primary care point of view as opposed to the public health perspective previously accomplished by the French Health Insurance. The robust design of this study included a sufficient number of patients therefore the lack of demonstrated effect is unlikely correlated to an insufficient power of the trial: if an effect exists, it is insufficient to be considered as clinically relevant, i.e. able to enhance substantially the seasonal influenza vaccination uptake. Through randomization groups were comparable from baseline to the end of the study. The generalized mixed linear statistical model with maximized likelihood adjustment gave a precise evaluation of the effect on the main outcomes.

A possible bias could involve the share of patients vaccinated directly by a nurse without being exposed to the GP intervention as some patients may have received the invitation to be vaccinated directly from the Health Insurance. Nonetheless, the present study has been randomized and the groups are being comparable leading to conclude that this lack of exposure to the intervention was comparable in both groups, without other influence on the result than limiting the impact of the intervention. Some GPs of the intervention group refused partially or totally to redesign their waiting rooms when the intervention was positioned. This barrier signs that the trial was performed in real live conditions. The intention to treat analysis of the outcomes considers this limit and no per-protocol analysis has been conducted. Another limitation is that vaccines provided by pharmacies have been considered as full vaccinations. The research group stated in the study protocol that the number of vaccines provided by pharmacies that were not injected to patients was negligible: this hypothesis is the usual one used by health authorities to investigate the vaccination coverage rate but has not been investigated.[1] However, the number of delivered and not injected vaccines is probably comparable in both arms of the trial. As patients under the age of 16 were not enlisted with a GP by the health insurance before 2017, no data were collected in patients under the age of 16 with a chronic disease (like asthma). Encounters with a different GP than the one by whom patients were enlisted was not taken into account.

The influenza immunization rate increased in both arms of the study compared to the prior year. Contemporarily, at regional and national level, the number of vaccinated people decreased. The Hawthorne effect, i.e. the trend of people to change their behaviour when they know that they are under observation, might have masked the effect of posters and pamphlets. [29] The study group carries out a new study to compare the control group in this study with a new control group enrolled *a posteriori* and measure the Hawthorne effect (Zelen Design [30]).

## Conclusion

The findings of the present trial do not support the efficacy of an educational prevention strategy based on posters and pamphlets in GP waiting rooms with regard to health behaviour change. Nonetheless, an important Hawthorne effect in this trial is suspected, and the next step of the study group will try to measure it.

## Supporting information

S1 Consort Checklist (DOC)

S1 Fig Figure: Trial profile / CONSORT flow chart. (TIF)

S1 Dataset (XLSX)

S1 Advice from Ethic Committee (PDF)
