# Supplementary figures and images for: Randomized controlled trial on promoting influenza vaccination in general practice waiting rooms
Source: PLoS One. 2018 Feb 9;13(2):e0192155. doi: 10.1371/journal.pone.0192155 (PMC5806862; doi:10.1371/journal.pone.0192155)

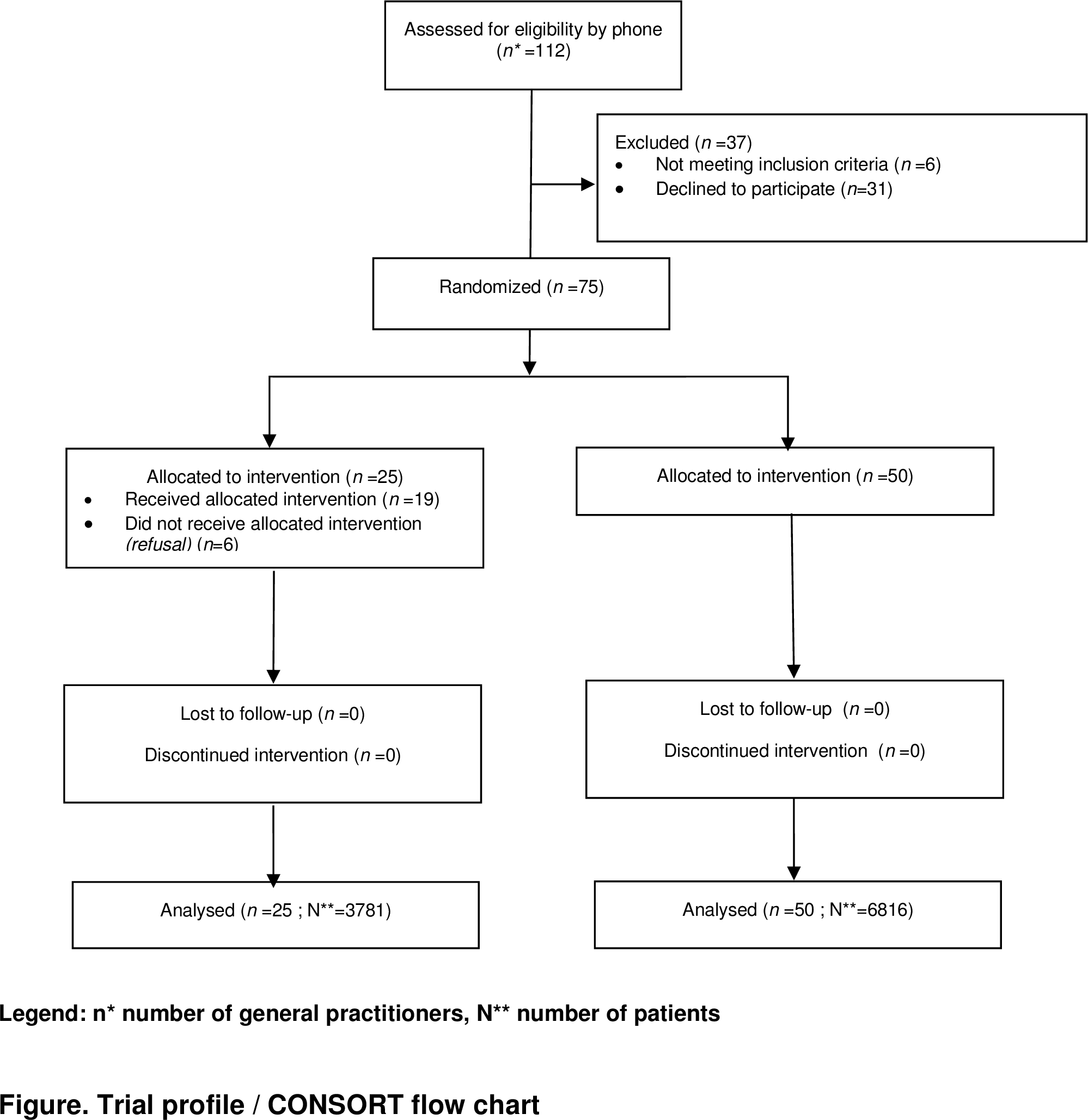

Supplement: S1 Fig — (TIF) [file pone.0192155.s002.tif]
