# Supplementary material for: Randomized controlled trial on promoting influenza vaccination in general practice waiting rooms
Source: PLoS One. 2018 Feb 9;13(2):e0192155. doi: 10.1371/journal.pone.0192155 (PMC5806862; doi:10.1371/journal.pone.0192155)
Supplement: S1 Advice from Ethic Committee — (PDF) [file pone.0192155.s004.pdf]

Bonjour,

Le numéro de dossier CPP est le HP 14/51.
Nous vous rappelons que dans notre avis nous vous avions mentionné de faire une déclaration auprès de la CNIL.

Bien cordialement.

Mme Marie-Noëlle Zimosz-Raux
Secrétariat CPP Nord Ouest IV
Bâtiment ex-USNB
6 rue du Professeur Laguesse
CHRU LILLE
CS 70001
59037 LILLE CEDEX

e-mail : cppnordouestiv@univ-lille2.fr
Permanence téléphonique de 13h30 à 16h30 chaque jour sauf le mercredi
Tel : 03.20.44.41.65
Fax : 03.20.44.41.63
http://www.comite-de-protection-des-personnes-nord-ouest-iv.sitew.fr/
